# Cancer gene expression profiles associated with clinical outcomes to chemotherapy treatments

**DOI:** 10.1186/s12920-020-00759-0

**Published:** 2020-09-18

**Authors:** Nicolas Borisov, Maxim Sorokin, Victor Tkachev, Andrew Garazha, Anton Buzdin

**Affiliations:** 1 Department of Bioinformatics and Molecular Networks, OmicsWay Corporation, Walnut, CA 91788 USA; 2 Moscow Institute of Physics and Technology, Dolgoprudny, Moscow Oblast 141701 Russia; 3 I.M. Sechenov First Moscow State Medical University (Sechenov University), Moscow, 119991 Russia; 4 Shemyakin-Ovchinnikov Institute of Bioorganic Chemistry, Moscow, 117997 Russia

**Keywords:** Machine learning, Transcriptomics, Gene expression, RNA sequencing, Microarrays, Molecular diagnostics, Biomarkers detection, Cancer, Clinical oncology, Personalized medicine, Chemotherapy

## Abstract

**Background:**

Machine learning (ML) methods still have limited applicability in personalized oncology due to low numbers of available clinically annotated molecular profiles. This doesn’t allow sufficient training of ML classifiers that could be used for improving molecular diagnostics.

**Methods:**

We reviewed published datasets of high throughput gene expression profiles corresponding to cancer patients with known responses on chemotherapy treatments. We browsed Gene Expression Omnibus (GEO), The Cancer Genome Atlas (TCGA) and Tumor Alterations Relevant for GEnomics-driven Therapy (TARGET) repositories.

**Results:**

We identified data collections suitable to build ML models for predicting responses on certain chemotherapeutic schemes. We identified 26 datasets, ranging from 41 till 508 cases per dataset. All the datasets identified were checked for ML applicability and robustness with leave-one-out cross validation. Twenty-three datasets were found suitable for using ML that had balanced numbers of treatment responder and non-responder cases.

**Conclusions:**

We collected a database of gene expression profiles associated with clinical responses on chemotherapy for 2786 individual cancer cases. Among them seven datasets included RNA sequencing data (for 645 cases) and the others – microarray expression profiles. The cases represented breast cancer, lung cancer, low-grade glioma, endothelial carcinoma, multiple myeloma, adult leukemia, pediatric leukemia and kidney tumors. Chemotherapeutics included taxanes, bortezomib, vincristine, trastuzumab, letrozole, tipifarnib, temozolomide, busulfan and cyclophosphamide.

## Background

Personalized approach provides important advantages in clinical oncology in terms of improved patient survival and lower drug toxicities [1, 2]. However, so far it can only cover a minor fraction of cancer patients [3, 4] due to lack of robust prognostic biomarkers for most of the treatments [5]. The proportion of patients eligible for personalized oncology slightly grows. For example, the percentage of US patients with cancer estimated to benefit from personalized prescriptions of targeted therapeutics was only 0.7% in 2006, and it had increased to ~ 5% in 2018 [4]. However, this progress could be more significant if more companion diagnostic tests would be available for the standardly used cancer drugs. In this regard, gene expression data, either obtained by RNA sequencing [1] or using microarrays [6], frequently provide an advantage over genomic tests. Several trials and clinical case reports were published recently evidencing high efficiency of gene expression-based prescriptions of cancer chemotherapeutics. Cancer gene expression data can be used per se or can be normalized on the available profiles of healthy human tissues [7].

Using transcriptomic data, bioinformatic models can be built for patient-oriented ranking of cancer drugs [8]. These models can be hypothesis-driven, e.g. based on the knowledge of the specific mechanisms of drugs anti-cancer activities [9–11]. Alternatively, hypothesis-free approaches like machine learning (ML) don’t need any theoretic background but instead strongly require sufficient training and validation datasets. Many ML methods may be used for such applications, e.g. decision trees [12, 13], random forests, RF [14, 15], linear [16], logistic [17], lasso [18, 19], ridge [15, 20] regressions, multi-layer perceptron, MLP [12, 15, 21, 22], support vectors machines [12, 13, 15, 23–25], adaptive boosting [26–28], as well as binomial naïve Bayesian [15] method.

High-quality training and validation datasets are required to run both types of the above models. Nowadays there is a shortage of clinically annotated molecular data that would help developing ML-assisted diagnostic tools. The datasets available are usually considered too small for applying ML [23, 25, 26, 29–33]. Indeed, the figure of dozens or hundreds of annotated biosamples is negligible in comparison with ~ 20,000 protein coding genes measured in transcriptomic assays. Intelligent data filtering is, therefore, needed to reduce dimensionality of data [8]. However, a recent approach using dynamic feature extraction, or flexible data trimming, can significantly improve performances of ML-based methods for the real-world datasets [15, 25].

This study was performed to review available clinically annotated datasets of cancer transcriptomic profiles that may be suitable for applications in ML models. To our knowledge, this is the largest published collection of processed gene expression data coupled with case history excerpts indicating positive or negative response to certain treatment protocols for cancer patients. This manually curated collection of molecular datasets will be helpful for those working with the ML or artificial intelligence applications in oncology, as well as for the fundamental research and development of cancer biomarkers.

## Methods

We curated GEO [34], TARGET [35] and TCGA [36] repositories to extract cancer gene expression profiles associated with the clinical outcomes of chemotherapeutic treatments. We attempted to build a knowledgebase of molecular datasets suitable for building ML classifiers of clinical responses on chemotherapy treatments (Table 1, Additional file 1). Every included dataset met the following criteria:

- at least 40 gene expression profiles present;
- data obtained for the same cancer type and using the same experimental platform
- every profile is linked with the case clinical history
- all cancers treated with at least one common drug or chemotherapy regimen
- treatment outcomes are available enabling to classify every case as either responder or non-responder.

**Table 1:** Overview of selected transcriptomic datasets of responders/non-responders to cancer chemotherapy, responders (R) vs non-responders (NR)

Reference	Dataset ID	Disease type	Therapy	Experimental platform	Number *N* of cases (R vs NR)	Number of *core marker genes (S)*
[37, 38]	GSE25066	Breast cancer with different hormonal and HER2 status	Neoadjuvant taxane + anthracycline	Affymetrix Human Genome U133 Array	508 (118 R: *complete response* + *partial response*; 389 NR: *residual disease + progressive disease*)	20
[39]	GSE41998	Breast cancer with different hormonal and HER2 status	Neoadjuvant doxorubicin + cyclophosphamide, followed by paclitaxel	Affymetrix Human Genome U133 Array	124 (90 R: *complete response* + *partial response*; 34 NR: *residual disease + progressive disease*)	11
[40]	GSE20271	Breast cancer with different hormonal and HER2 status	Paclitaxel + fluorouracil + adriamycin + cyclophosphamide	Affymetrix Human Genome U133A Array	85 (18 R: *complete response* + *partial response*; 66 NR: *residual disease + progressive disease*)	11
[41]	GSE50948	Breast cancer with different hormonal and HER2 status	Paclitaxel + doxorubincin followed by cyclophos-phamide + methotrexate/ fluorouracil followed by trastuzumab	Affymetrix Human Genome U133 Plus 2.0 Array	156 (53 R: *complete response* + *partial response*; 103 NR: *residual disease + progressive disease*)	19
[42]	GSE9782	Multiple myeloma	Bortezomib monotherapy	Affymetrix Human Genome U133 Array	169 (85 R: *complete response* + *partial response*; 84 NR: *no change* + *progressive disease*)	18
[43]	GSE39754	Multiple myeloma	Vincristine + adriamycin + dexamethasone followed by autologous stem cell transplantation (ASCT)	Affymetrix Human Exon 1.0 ST Array	136 (74 R: *complete*, *near-complete* and *very good partial responders*; 62 NR: *partial*, *minor* and *worse*)	16
[44]	GSE68871	Multiple myeloma	Bortezomib-thalidomide-dexamethasone	Affymetrix Human Genome U133 Plus	118 (69 R: *complete*, *near-complete* and *very good partial responders*; 49 NR: *partial*, *minor* and *worse*)	12
[45]	GSE55145	Multiple myeloma	Bortezomib followed by ASCT	Affymetrix Human Exon 1.0 ST Array	61 (33 R: *complete*, *near-complete* and *very good partial responders*; 28 R: *partial*, *minor* and *worse*)	14
[35, 46]	TARGET-50	Childhood kidney Wilms tumor	Vincristine sulfate + cyclosporine, cytarabine, daunorubicin + conventional surgery + radiation therapy	Illumina HiSeq 2000	122 (36 R: *complete*, *near-complete* and *very good partial responders*; 86 NR: *partial*, *minor* and *worse*)	14
[35, 47]	TARGET-10	Childhood B acute lymphoblastic leukemia	Vincristine sulfate + carboplatin, cyclophosphamide, doxorubicin	Illumina HiSeq 2000	98 (30 R, 68 NR: see Fig. 1)	14
[35]	TARGET-20	Childhood acute myeloid leukemia	Non-target drugs (asparaginase, cyclosporine, cytarabine, daunorubicin, etoposide; methotrexate, mitoxantrone) including busulfan and cyclo-phosphamide	Illumina HiSeq 2000	54 (31 R, 23 NR: see Fig. 1)	10
[35]	TARGET-20	Childhood acute myeloid leukemia	Same non-target drugs, but excluding busulfan and cyclo- phosphamide	Illumina HiSeq 2000	142 (62 R, 80 NR: see Fig. 1)	16
Reference	Dataset ID	Disease type	Therapy	Experimental platform	Number *NC* of cases (R vs NR)	Number of *core marker genes (NS)*
[48]	GSE18728	Breast cancer	Docetaxel, capecitabine	Affymetrix Human Genome U133 Plus 2.0 Array	61 (23R: *complete response* + *partial response*; 38 NR: *residual disease + progressive disease*)	16
[49]	GSE20181	Breast cancer	Letrozole	Affymetrix Human Genome U133A Array	52 (37 R: *complete response* + *partial response*; 15 NR: *residual disease + progressive disease*)	11
[50]	GSE20194	Breast cancer	Paclitaxel; (tri) luoroacetyl chloride; 5-fluorouracil, epirubicin, cyclophosphamide	Affymetrix Human Genome U133A Array	52 (11 R: *complete response* + *partial response*; 41 NR: *residual disease + progressive disease*)	10
[51]	GSE23988	Breast cancer	Docetaxel, capecitabine	Affymetrix Human Genome U133A Array	61 (20 R: *complete response* + *partial response*; 41 NR: *residual disease + progressive disease*)	18
[52]	GSE22358	Breast cancer	Docetaxel, capecitabine	Agilent UNC Perou Lab *Homo sapiens* 1X44K Custom Array	122 (116 R: *complete response* + *partial response*; 6 NR: *residual disease + progressive disease*)	2
[53]	GSE32646	Breast cancer	Paclitaxel, 5-fluorouracil, epirubicin, cyclophosphamide	Affymetrix Human Genome U133 Plus 2.0 Array	115 (27 R: *complete response* + *partial response*; 88 NR: *residual disease + progressive disease*)	17
[54]	GSE37946	Breast cancer	Trastuzumab	Affymetrix Human Genome U133A Array	50 (27 R: *complete response* + *partial response*; 23 NR: *residual disease + progressive disease*)	14
[55]	GSE42822	Breast cancer	Docetaxel, 5-fluorouracil, epirubicin, cyclophosphamide, capecitabine	Affymetrix Human Genome U133A Array	91 (38 R: *complete response* + *partial response*; 53 NR: *residual disease + progressive disease*)	13
[56]	GSE5122	Acute myeloid leukemia	Tipifarnib	Affymetrix Human Genome U133A Array	57 (13 R: *complete response* + *partial response* + *stable disease*; 44 R: *progressive disease*)	10
[57]	GSE59515	Breast cancer	Letrozole	Illumina HumanHT-12 V4.0 expression beadchip	75 (51 R: *complete response* + *partial response*; 24 NR: *residual disease + progressive disease*)	15
[58]	GSE76360	Breast cancer	Trastuzumab	Illumina HumanHT-12 V3.0 expression beadchip	48 (42 R: *complete response* + *partial response*; 6 NR: *residual disease + progressive disease*)	3
[36]	TCGA-LGG	Low-grade glioma	Temozolomide + (optionally) mibefradil	Illumina HiSeq 2000	131 (100 R: *complete response* + *partial response* + *stable disease*; 31 NR: *progressive disease*)	9
[36]	TCGA-LC	Lung cancer all types	Paclitaxel + (optionally), cisplatin/carboplatin, reolysin	Illumina HiSeq 2000	41 (24 R: *complete response* + *partial response* + *stable disease*; 17 NR: *progressive disease*)	7
[36]	TCGA-UC	Uterine corpus endothelial carcinoma	Paclitaxel + (optionally) carboplatin, cisplatin, doxorubicin	Illumina HiSeq 2000	57 (57 R: *complete response* + *partial response* + *stable disease*; 7 NR: *progressive disease*)	2

We used different approaches to discriminate between the treatment responders and non-responders. Where available, e.g. for the datasets extracted from the GEO repository, we used the responder/non-responder marks assigned by the authors of the original communications publishing these data. In many instances, the number of response groups was more than two and included groups like “partial responders”. However, most frequently binary ML-assisted drug response classifiers are needed that classify patients in only two classes: either responders or non-responders [8, 23, 25, 29, 30].

If a binary classifier is needed, then the number of clinical response groups in the training/validation datasets must be also condensed to two, i.e. responders and non-responders. In such case, the groups identified by the authors as *partial responders* probably can be combined with the responders. This is the case for all current breast cancer datasets, namely GSE25066 [37, 38], GSE41998 [39], GSE20271 [40], GSE50948 [41], GSE18728 [48], GSE20181 [49, 59], GSE20194 [50], GSE23988 [51], GSE22358 [52], GSE32646 [53], GSE37946 [54], GSE42822 [55], GSE59515 [57] and GSE76360 [58].

For the TCGA profiles, namely for the low-grade glioma (TCGA-LGG), lung cancer (TCGA-LC), and uterine corpus endothelial carcinoma (TCGA-UEC) datasets, and for the acute myeloid leukemia dataset GSE5122 [56], *stable disease* cases can be most probably classified as the responders whereas *progressive disease* cases – as the non-responders. For the multiple myeloma dataset GSE9782 [42], the classification can be used as defined by the authors, where patents with *complete* and *partial response* were annotated as the responders, and with *no change* and *progressive disease* – as the non-responders. For three other multiple myeloma datasets, namely GSE39753 [43], GSE68871 [44], and GSE55145 [45], *complete*, *near-complete* and *very good partial response* groups can be most likely considered as the responders, whereas *partial*, *minor* and *worse response* groups – as the non-responders.

**Fig. 1:**
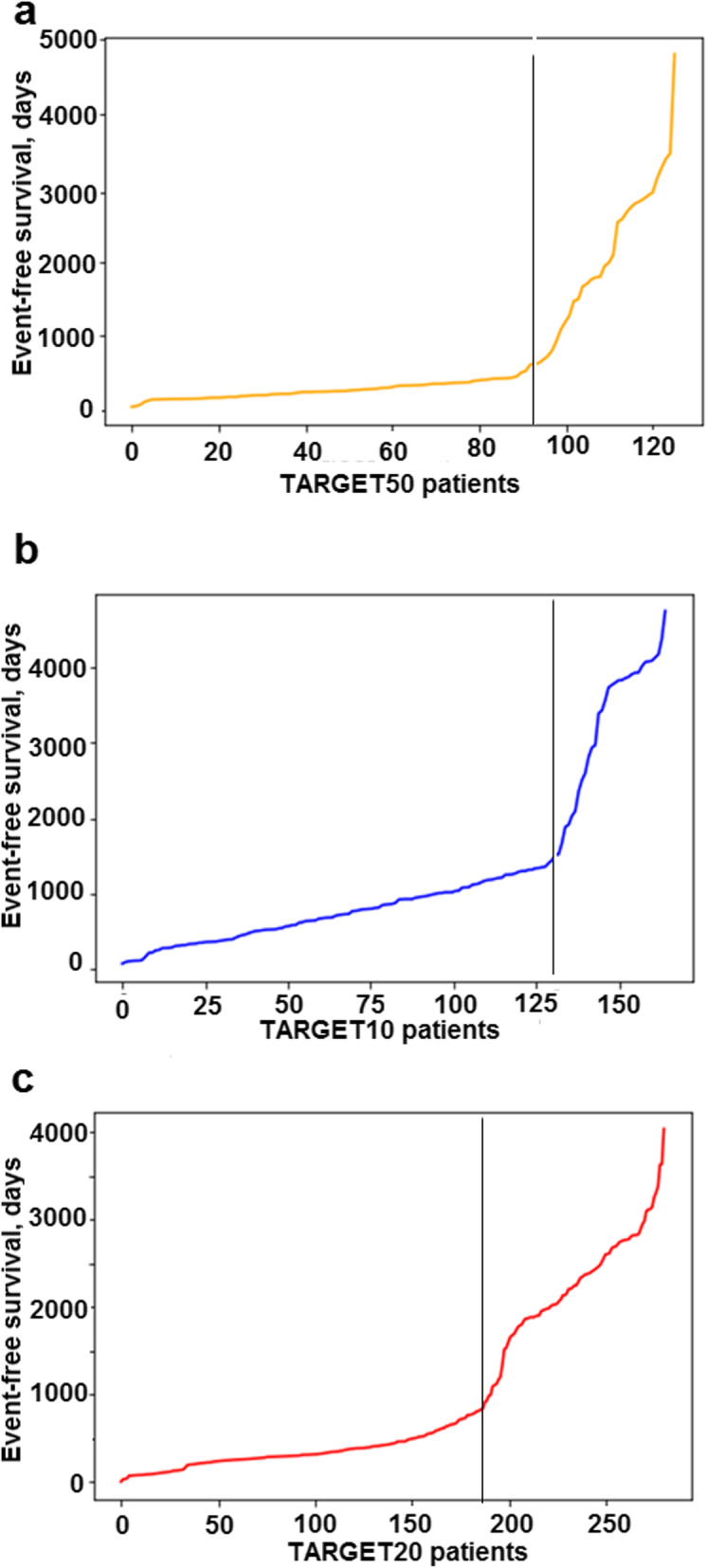
Distribution of event-free survival time for the patients with (**a**) childhood kidney Wilms tumor from TARGET-50 dataset, (**b**) childhood ALL from TARGET-10 dataset and (**c**) childhood AML from TARGET-20 dataset [35]. Patients on the left from vertical threshold can be considered as the non-responders, and on the right – as the responders to the treatment

Classification of the TARGET repository profiles was more sophisticated as no responder classification was given by the authors. This was the case for the datasets of pediatric Wilms kidney tumor (TARGET-50), acute myeloid leukemia (TARGET-20) and acute lymphoblastic leukemia (TARGET-10) extracted from the gene expression repository of National Cancer Institute [35]. However, these latter clinical cases were annotated by the time of event-free survival. Distributions of the event-free survival time enabled us to identify for every dataset two different modes of survival with different slopes (Fig. 1), that can be recognized as either responders or non-responders.

## Results

**Fig. 2:**
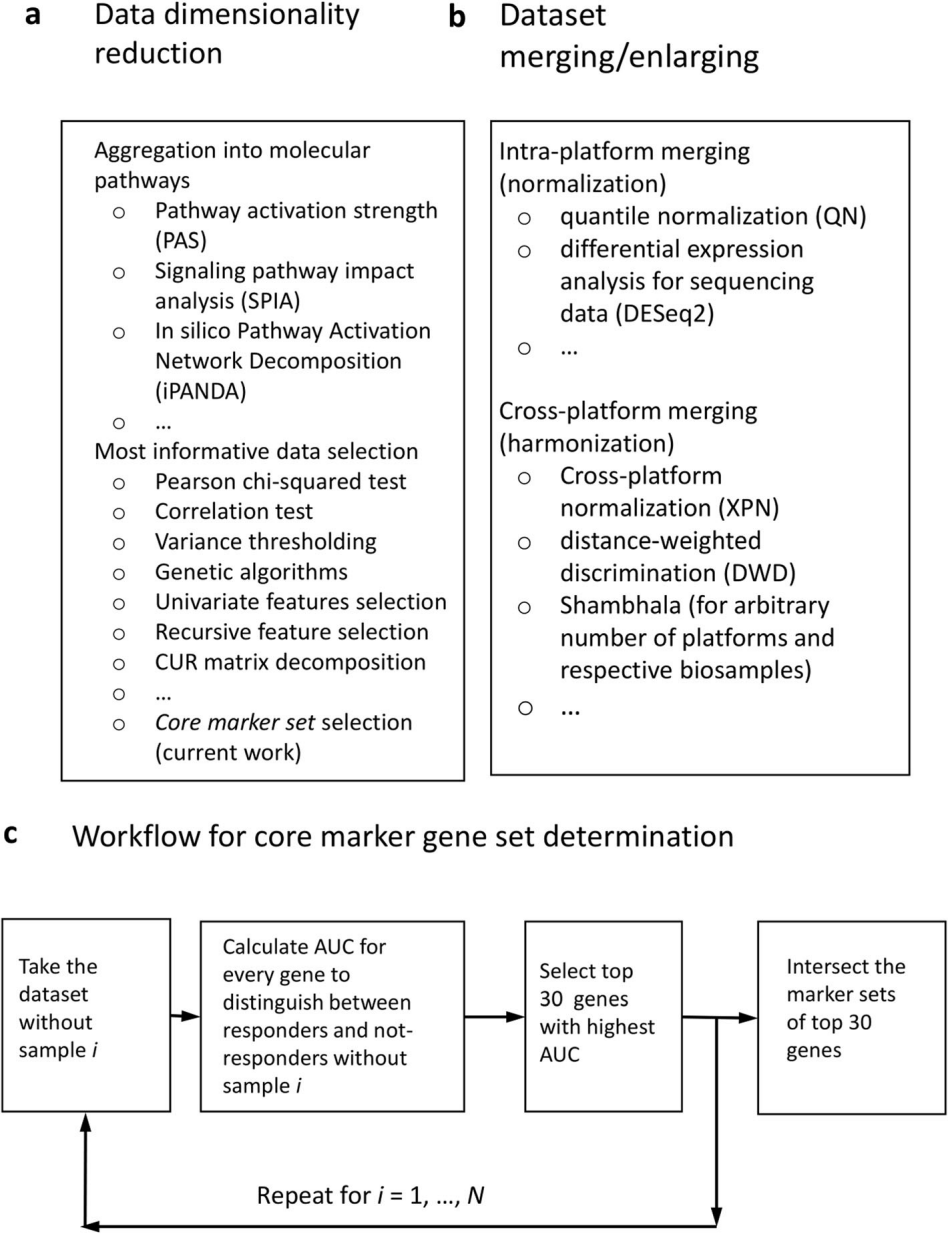
Possible scenarios of using ML to build classifiers based on gene expression datasets. **a** Methods data dimensionality reduction; **b** approaches to merging and enlarging of gene expression datasets for ML application; **c** general workflow for a core marker set determination

For raw gene expression data, the number of features i.e. interrogated genes, usually exceeds the number of tumor cases by roughly two orders of magnitude. Therefore, for robust application of ML the dimensionality of data must be reduced to make the number of selected features lower than the number of tumor cases or at least comparable to it (Fig. 2a). To reduce dimensionality, the gene expression data can be aggregated into the higher-order molecular markers like activation profiles of molecular pathways [23, 29, 30, 60, 61]. Alternatively, the most informative fraction of the initial data can be selected that can distinguish between the responder and non-responder classes. For selection of such marker features, several approaches have been proposed, e.g. Pearson chi-squared test [62], correlation test [27, 62], variance thresholding, genetic algorithms [63], univariate feature selection, recursive feature elimination, principal component analysis [27], CUR matrix [64], decomposition [65] and covariate regression [66].

In the current research, we applied the following leave-one-out-based method for finding robust marker features [25] (Fig. 2c). Imagine that we have a gene expression dataset that embraces *N* clinical cases, each with corresponding expression profile. For each clinical case *i* = 1, … *N*, we determine the top *Q* marker genes that distinguish responding and non-responding cases in a *sub-dataset* that contains all samples but *i*. In other words, for all *N sub-datasets* each having *N*-1 cases, we interrogate each gene taken one by one and retrieve the top *Q* set of genes that showed the highest ROC AUC values for the difference between responder and non-responder profiles. The quality metric *area under the ROC curve* (AUC) is the universal metric of a biomarker robustness that depends on its sensitivity and specificity [67]. It positively correlates with the quality of a biomarker and varies from 0.5 till 1. The standard discrimination threshold is 0.7 and the entries with higher AUC are considered high-quality biomarkers, and vice versa [68]. AUC is broadly used for detection of biomarkers in oncology [69–73].

To provide trobust feature selection, the number *Q* shouldn’t exceed the number of cases *N*. In the current application, we took *Q* equal to 30 because all tdatasets under consideration had more than 40 cases. The final list of core marker genes was obtained by intersecting top *Q* gene sets for all *N* sub-datasets.

We applied this procedure to all the clinically annotated cancer transcriptomic datasets under consideration and identified for them *core marker genes* (Table 1). Twenty-three out of 26 datasets investigated provided 7–20 core marker gene features for further ML applications (Table 1).

The remaining three datasets, namely GSE22358 [52], GSE76360 [58] and TCGA-UEC [36], were poorly balanced because the numbers of responders greatly exceeded the respective numbers of non-responders, or vice versa. For these three instances we were unable to generate robust core marker gene sets for ML applications because the number of such genes was too low (two-three per dataset, Table 1).

## Discussion

By the current moment, ML hasn’t made a revolution in biomedicine [12]. This may be partly connected with the relatively recent emergence of experimental methods generating big amounts of biomedical data combined with the developed IT infrastructure. Among these game-changing methods the major role was played by the next-generation sequencing (NGS) and novel mass-spectrometry approaches which made whole genome-, transcriptome-, proteome- and metabolome analyses relatively fast and cheap [74–76].

Yet further development of ML methods in personalized oncology is still strongly limited by the low number of clinically annotated cancer patient molecular datasets. A dataset suitable for ML should have all together enough number of high-throughput molecular profiles and also the associated clinical case history records featuring success of the therapeutic regimen used.

In this paper we reviewed three major repositories of omics data for the available responder/non-responder datasets including more the 40 cancer cases treated with the same chemotherapeutics. We identified 26 datasets with totally 2786 cases, ranging from 41 till 508 cases per dataset (Table 1). We checked the robustness of these datasets and their suitability for ML applications using our previous method of core maker feature determination [25]. According to this test, 23/26 datasets were suitable for ML, each having 7–20 core marker genes/features for further ML applications. Contrarily, the remaining three datasets produced only two or three features, which may seem insufficient for the ML. Poor performance of these three datasets was most likely due to unbalanced numbers of clinical responder/non-responder cases included.

To increase the number of cases (Fig. 2b), the datasets for the same disease or drug treatment conditions can be merged using cross-dataset harmonization. Different methods can be used to harmonize data obtained using the same [77, 78] or two different experimental platforms [79, 80], or even using multiple platforms [81] (Fig. 2b).

In addition, when the cases are deficient, *transfer learning* methods may be used for a certain disease or drug condition. Using this approach, the ML training process may be preformed on the multiple available molecular profiles corresponding to cell culture treated with certain drugs [82], whereas the ML classifier validation may be done on more rare patient cancer cases [23, 29, 30].

## Conclusions

We identified 26 clinically annotated gene expression datasets ranging from 41 till 508 cases per dataset (Table 1). Collectively, they covered 2786 individual cancer cases. Among them seven datasets included RNA sequencing data (for 645 cases) and the others – microarray expression profiles. The datasets represented breast cancer, lung cancer, low-grade glioma, endothelial carcinoma, multiple myeloma, adult leukemia, pediatric leukemia and kidney tumors. Chemotherapeutics used included taxanes, bortezomib, vincristine, trastuzumab, letrozole, tipifarnib, temozolomide, busulfan and cyclophosphamide.

We hope that presented collection of clinically annotated transcriptomic profiles will be useful to those working with data analysis in oncology, as well as for the fundamental research and development of next-generation cancer biomarkers.

## Supplementary information

**Additional file 1.** Clinically annotated datasets and samples they contain.

## Data Availability

All the data, including IDs of expression profiles, treatment methods and clinical response assessment, both done by the teams, who had worked with them, and our binary (“P-vs-N”) response classifications, are provided in Additional file 1.

## Abbreviations

ALL: Acute lymphoblastic leukemia
AML: Acute myelogenous leukemia
ASCT: Autologous stem cell transplantation
GEO: Gene expression omnibus
GSE: GEO series
LOO: Leave-one-out
ML: Machine learning
MLP: Multi-layer perceptron
PM: Personalized medicine
RF: Random forest
ROC: Receiver operating characteristic
SVM: Support vector machines
TARGET: Tumor Alterations Relevant for GEnomics-driven Therapy
TCGA: The Cancer Genome Atlas
